# Relationship between HPV and the biomarkers annexin A1 and p53 in oropharyngeal cancer

**DOI:** 10.1186/1750-9378-9-13

**Published:** 2014-04-21

**Authors:** Cleberson Jean dos Santos Queiroz, Cíntia Mara de Amorim Gomes Nakata, Egle Solito, Amílcar Sabino Damazo

**Affiliations:** 1 Post-Graduation in Health Science, Faculty of Medicine (FM), Federal University of Mato Grosso (UFMT), Mato Grosso, MT 78060-900, Brazil; 2 William Harvey Research Institute; Barts and The London School of Medicine and Dentistry, Queen Mary University of London, London EC1M 6BQ, UK; 3 Department of Basic Science in Health; Faculty of Medicine (FM), Federal University of Mato Grosso (UFMT), Mato Grosso, MT 78060-900, Brazil; 4 Department of Gastroenterology, Institute of Translational Medicine, University of Liverpool, Liverpool L69 3GE, UK; 5 Henry Wellcome Laboratory, University of Liverpool, 1st Floor, Nuffield Building, Liverpool L69 3GE, UK

**Keywords:** Annexin A1, HPV, p53, Cancer, Oropharynx

## Abstract

**Background:**

Human papillomavirus (HPV) is often present in oropharyngeal cancers. Head and neck tumors have been examined for other molecular markers including p53 and annexin A1 (ANXA1). Here, we investigated the prevalence of HPV and its relationship with p53 and ANXA1 in patients with oropharyngeal cancer.

**Methods:**

We have analyzed tumor and adjacent mucosa from 22 patients with squamous cell carcinoma of the oropharynx in addition to samples of the oropharyngeal epithelium in subjects without cancer. We evaluated the presence of the HPV (subtypes 16/18 and 31/33) by chromogenic *in situ* hybridization. Additionally, we used immunofluorescence to examine the expression of p16, p53, ANXA1 and the phosphorylation of the ANXA1 residues Ser27 (ANXA1-SER) and Tyr21 (ANXA1-TYR).

**Results:**

We have detected the presence of HPV genome in 59% of the 22 tumors. Of those, 92% were also positive for p16 immunostaining. Furthermore, we demonstrated a reduction in the expression of p53 in HPV + compared to HPV- tumors. Also, a reduction was observed in the expression of ANXA1 in tumors compared to epithelium from the margins and from controls. We also noted a reduction in ANXA1-TYR in tumors. However, the expression of both ANXA1 and ANXA1-SER were elevated in the margins of the HPV + *versus* HPV- tumors.

**Conclusions:**

Our results confirm a high prevalence of HPV in oropharyngeal cancer and a reduction in p53 expression in HPV + tumors. We observed a hypoexpression of ANXA1 and ANXA1-TYR in oropharyngeal cancer. The increase in ANXA1-SER in the margins of HPV + tumors suggests that the epithelium in these cases had been activated by an infectious agent. Those findings indicate that ANXA1 and its phosphorylated forms can play important roles in the response to HPV infection and the carcinogenesis of the oropharynx.

## Background

Cancers of the head and neck are a global health problem. These tumors occur frequently, especially among low-income populations, with a high social cost because of the associated morbi-mortality. Annually, this pathology affects approximately 633,000 people worldwide causing 325,000 deaths [1]. The main risk factors are smoking and consumption of alcohol [2]. However, a group of patients exists that lack these precursors, leading to the suspicion that other factors might play important roles in the process of carcinogenesis in this region [3,4]. The discovery of biomarkers that could ultimately translate into prognostic or predictive tools as well as possible new drug targets is, therefore, an important medical need.

Cancer of the oropharynx stands out from other topographies in the head and neck region due to its strong association with the infection caused by human papillomavirus (HPV). Currently, possible changes to the therapeutic approach based on HPV status are under discussion because of evidence suggesting that the presence or absence of the virus leads to distinct biological mechanisms and behaviors of the tumor [5]. HPV is an epitheliotropic virus associated with several cutaneous and mucosal pathological conditions, both benign and malignant. Various studies have demonstrated the presence of high-risk carcinogenic subtypes (especially subtypes 16, 18, 31 and 33) in a substantial proportion of these tumors [6]. Recent studies have shown high infection rates through the demonstration of the presence of viral genome [5,7,8] or using immunostaining directed to p16, a protein known to be upregulated in the presence of high-risk HPVs and frequently used as a surrogate of HPV infection [7,9,10].

Tumor suppressor protein p53 has several functions that are related to maintaining genomic stability and inhibiting cell proliferation in response to DNA damage. For preventing neoplasias to occur, the most important of these functions are cessation of cell growth [11,12] and induction of either apoptosis [13,14] or senescence [15,16]. P53 performs these functions both by acting as a transcription factor (inhibiting or stimulating the transcription of specific genes) and by acting directly on various elements of the regulatory machinery of the cell cycle [17]. Under normal conditions, the protein is expressed only in small quantities and with a very short half-life (only few minutes) and is normally undetectable by methods such as immunofluorescence. When mutated or inactivated, it becomes unable to impede the progression of the carcinogenic process and begins to accumulate in cells and, thus, can be easily identifiable [18].

Annexin-A1 (ANXA1) is a 37-kDa protein that is present in various cell types and belongs to the annexin family (proteins that share the capacity to bind in a calcium-dependent manner with the phospholipids that are present in negatively charged membranes) [19]. Evidence indicates that ANXA1 participates in signal transduction [20], in inflammation [19,21-23], in controlling cell growth and apoptosis [24,25], and in endocytosis [26], among other functions. In addition to the general properties attributed to ANXA1, post-translational changes such as phosphorylation are known to have an important influence on the activities performed by this protein [27-31].

Various types of cancers induce a differential expression of ANXA1. In breast cancer, the loss of ANXA1 expression is associated with poorer global survival rates [32]. In patients with gastric cancer, evidence shows that the loss of ANXA1 is associated with tumor progression, lymph node metastases, and poor prognosis [33]. A reduction in ANXA1 expression has been found in prostate cancer compared to non-tumor tissue [34]. On the other hand, an elevated expression of ANXA1 has been demonstrated in hepatocellular cancer [35], pancreatic cancer [36], glial tumors [37], and even in breast cancer [38], reinforcing the contradictory findings to date. In adenocarcinomas of the esophagus and the esophagogastric region, the hyperexpression of ANXA1 is associated with a poorer prognosis than that for tumors of this histology with a low expression of the protein [39]. Evidence indicates that ANXA1 that is phosphorylated at the tyrosine 21 residue (ANXA1-TYR) is present in higher levels in cervical cancer than in normal tissues [27].

Based on the data described above, our hypothesis is that ANXA1 and its phosphorylated forms could be differentially expressed in oropharyngeal cancer and that a relationship might exist between its expression, the expression of p53 and the presence of HPV infection. Therefore, we evaluated the presence of HPV infection (serotypes 16/18 and 31/33) in samples of squamous cell carcinoma of the oropharynx and analyzed the expression, through immunofluorescence (IF), of p16, p53, ANXA1 and two phosphorylated forms of ANXA1 (at specific serine and tyrosine residues). Based on the results that we obtained, we assessed the potential relationship among these markers.

## Results

### Histopathological tumor analysis

The evaluation of the tumors with hematoxylin and eosin staining confirmed that all the cases consisted of squamous cell carcinomas. In the tumor samples analyzed, we observed 5 (22.7%) well-differentiated, 6 (27.3%) moderately differentiated, and 10 (45.5%) poorly differentiated carcinomas. One (4.5%) of the samples was very small and did not allow for histological grading. No significant relationship was observed between the level of ANXA1 expression and the grade of tumor differentiation.

### Characterization of the presence of HPV and the expression of p16 and p53

**Figure 1:**
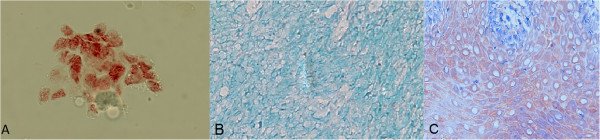
**Chromogenic *in situ *hybridization for detection of HPV 16/18. A)** Positive control (CasKi cells, magnification: 600x); **B)** Negative cancer sample (400x); **C)** Positive cancer sample (400x).

The analysis of HPV infection by CISH (Figure 1) indicated the presence of the viral DNA in 13 (59.1%) patients. All of the HPV + cases showed positivity for the probe combination targeting serotypes 16/18 (meaning that these samples might be positive for HPV16, HPV18 or both). Two cases were positive for both the 16/18 probe mix and the 31/33 probe mix, while no cases were positive for the 31/33 probe mix alone. Among the HPV + cases, the proportion of males was 61%, whereas in the HPV- group, 100% of the cases were males (*p* = 0.05). We did not observe significant differences in terms of age and the presence of the risk factors of smoking and drinking between HPV + and HPV- groups (Table 1). None of the 9 HPV-negative carcinomas (based on CISH) showed positive staining for p16 through IF. On the other hand, 12 out of 13 HPV-positive samples were positive for p16 staining (92.3%). Therefore, we used the CISH-positive set for the comparisons with the other markers.

**Table 1:** Patient characteristics according to HPV status

**Characteristics**	**HPV+**	**HPV-**	***p*-value**
Age			ns
Average	53.1	56.9	
Range	38 – 79	46 – 69	
Sex			0.05
Male	61%	100%	
Female	39%	0%	
Smoking	86%	100%	ns
Alcohol consumption	29%	50%	ns

ns = not significant.

**Figure 2:**
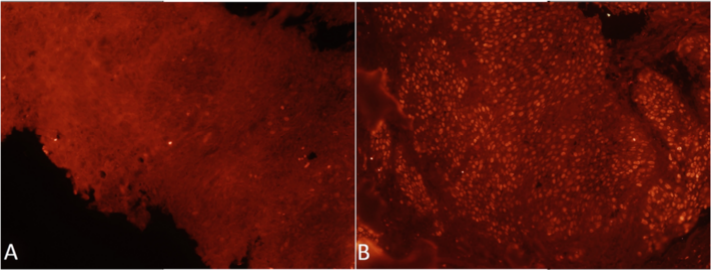
**Nuclear expression of the p53 protein in oropharyngeal cancer. A)** HPV-positive cancer sample exhibiting absence of p53 immunofluorescence. **B)** HPV-negative cancer sample exhibiting nuclear fluorescence denoting expression of the accumulated protein in tumor tissue. Magnification: 400x.

Using IF (Figure 2), we analyzed the nuclear expression of the p53 protein in 21 of the 22 samples (one HPV + case did not undergo this analysis for technical reasons). We demonstrated nuclear positivity for p53 in 7 (33.3%) of the 21 samples. Of the 12 HPV + samples that were tested for p53, only 2 (16.6%) expressed p53, while of the 9 HPV- samples, 5 (55%) expressed the protein (*p* < 0.001).

### Analysis of ANXA1 expression in oropharyngeal cancer

**Figure 3:**
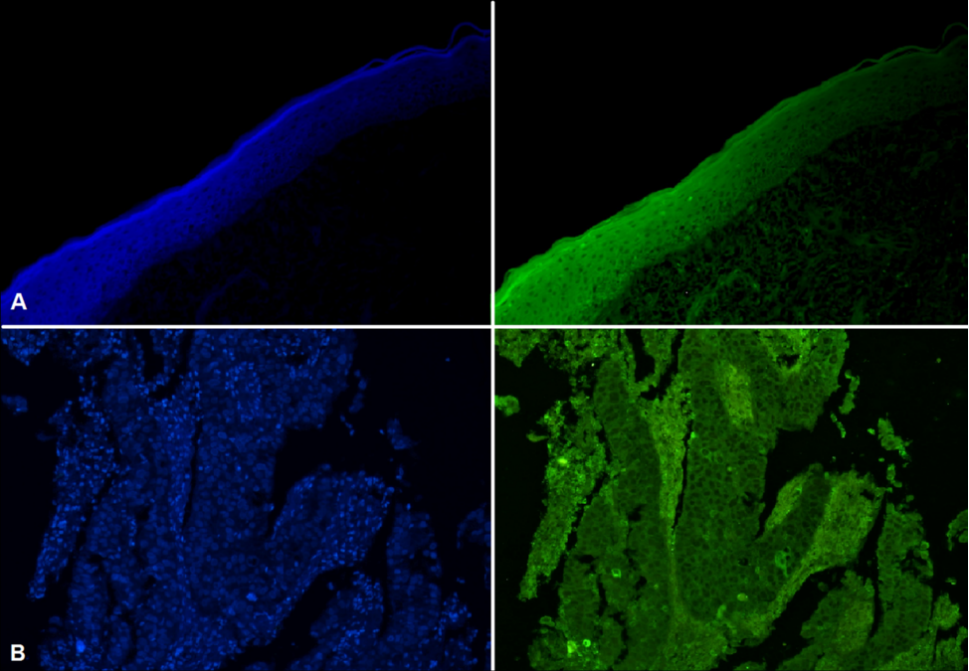
**Expression of ANXA1 protein. A)** Epithelial adjacent margin. **B)** Oropharyngeal squamous cell carcinoma. Left: nuclei stained by DAPI (blue) to enable architectural definition. Right: same sections as in left, showing cytoplasmic ANXA1 expression (green). Note the lower expression of ANXA1 in B (right) compared to its stromal tissue and to normal epithelium in A (right). Magnification: 200x.

We initially analyzed ANXA1 expression on the epithelial margin of the tumors and in the epithelia of the non-neoplastic controls, and we did not observe any significant differences between the groups. We then made comparisons between the tumor tissues and both groups of epithelia (margins and non-neoplastic controls) (Figure 3). In addition, we analyzed the expression of ANXA1 according to the status of p53 (p53+ *vs.* p53-) and the presence or absence of HPV. We observed a global reduction in the expression of ANXA1 in the neoplastic tissues compared to that in the epithelia of both margins and controls (Table 2). This ANXA1 hypoexpression in the tumors was evident in both the HPV + and HPV- cases, except in the group of HPV- and p53- tumors. Analysis of ANXA1 expression in the epithelial margins revealed an increase in ANXA1 expression in HPV + cases compared to that in the HPV- cases and the non-neoplastic controls. We found no significant difference in ANXA1 expression between p53+ and p53- tumors or between HPV + and HPV- tumors.

**Table 2:** Expression of ANXA1 according to HPV and p53 status

	**HPV+**		**HPV-**		
	**p53+**	**p53-**	**p53+**	**p53-**	**Controls**
Cancer	47.3 ± 2.7*	55.4 ± 5.7*	46.4 ± 4.5*	53.4 ± 3.0	-
Epithelium	105.0 ± 2.9^##^	107.5 ± 4.3^###^	79.2 ± 3.7	62.5 ± 2.5	82.3 ± 1.5

We quantified the ANXA1 protein expression in arbitrary units (a.u.) by determining the median optical density (MOD). The results are presented as the means ± SEM.

*p < 0.05 for cancer *versus* epithelium.

## *p* < 0.01; ### *p* < 0.001 for HPV + *versus* HPV-.

To better understand the role of ANXA1 in cancer of the oropharynx, we evaluated the expression of its phosphorylated forms in the tissues studied (Table 3). The expression of ANXA1-SER was similar between the tumors and the epithelia in the HPV- samples. However, in the HPV + cases, we noted an increase in its expression in the margin compared to the expression in both the neoplastic tissue and the controls. Expression of ANXA1-SER was similar between HPV + and HPV- groups.

**Table 3:** Expression of the phosphorylated forms of ANXA1

	**ANXA1-SER**			**ANXA1-TYR**		
	**HPV+**	**HPV-**	**Controls**	**HPV+**	**HPV-**	**Controls**
Cancer	13.5 ± 1.6	14.6 ± 0.4	-	15.3 ± 0.6**	13.6 ± 1.6*	-
Epithelium	23.2 ± 1.7*	16.0 ± 1.0	15.3 ± 0.3	22.2 ± 0.7	18.5 ± 1.5	26.7 ± 0.9

The results are described as the means ± SEM.

*p < 0.05 for epithelium *versus* cancer.

**p < 0.01 for epithelium *versus* cancer.

In contrast, the analysis of ANXA1-TYR expression revealed a significant reduction in cancerous tissues compared to the margin epithelium in both the HPV + and HPV- cases. No differences were observed in the expression of ANXA1-TYR between the HPV + and HPV- groups.

## Discussion

Our results have indicated presence of HPV in 59.1% of the cases of oropharyngeal cancer that were examined; this finding is similar to those reported in recently published studies [5,8]. It confirms the notion that viral DNA is often present in this pathology, independent of the geographical area studied. In all cases in which HPV was detected, the probe mix targeting serotypes 16 and 18 was positive. Since several studies have shown that HPV 16 is responsible for more than 90% of HPV-related oropharyngeal cancers [8,40], it is highly likely that the majority of our cases are linked HPV16.

We have noticed a reduced expression of ANXA1 in oropharyngeal cancer compared to the expression observed in the epithelium of the tumor margin and in the non-neoplastic controls, a finding that is in agreement with the majority of studies focusing on cancers of the head and neck. This pattern was observed both in HPV + cases and HPV- cases in which p53 was expressed. In the group that tested negative for both HPV and p53, no difference was observed in the expression of ANXA1 between the tumor and the normal epithelium. However, the absence of significant difference between these cases may be due to the limited sample size.

ANXA1, a protein related to inflammatory response and involved in cell proliferation, has been studied in various cancers with contrasting findings. In tumors of the head and neck area, studies have been done evaluating the role of ANXA1 in tumorigenesis, cellular differentiation and prognosis. In one study, researchers analyzed various proteins with potential to be biomarkers for differentiation and prognosis in patients with nasopharyngeal cancer [41]. The authors identified 36 proteins with differentiated expression between normal epithelium of the nasopharynx and nasopharyngeal carcinoma. Of these proteins, only 3 were considered to be potential biomarkers for carcinomas: stathmin (increased expression), 14-3-3ϭ (reduced expression), and ANXA1 (reduced expression). Various other studies have corroborated these findings, showing a reduction of ANXA1 expression in squamous cell carcinomas of the head and neck and revealing a relationship between this reduction and a lack of differentiation in the tumors [42-45]. To the best of our knowledge, the present study is the first to evaluate the expression of ANXA1 in association with HPV infection in oropharyngeal cancer, thereby demonstrating a relationship between two important and current markers for this disease.

Recently, a study was published evaluating protein and mRNA expression of ANXA1 in relation to the HPV status in patients with squamous cell carcinoma of the penis [46]. This study demonstrated an increase in ANXA1 expression in HPV + carcinomas compared to the expression detected in HPV- carcinomas. The authors’ proposed hypothesis was that the E6 viral oncoprotein may induce ANXA1 expression, an idea that had been suggested in a previous study [47]. In the present study, we observed an increase in ANXA1 expression in HPV + cases compared to HPV- cases only in normal epithelium of the tumor margin. This finding suggests the possibility that ANXA1 expression may be induced by the E6 protein as a precursor event prior to the malignant conversion of the infected cell. This inducer effect of ANXA1 expression may be lost in a clearly neoplastic cell from oropharyngeal site. However, we have not tested directly the normal epithelium for HPV, but the cancer counterpart. Therefore, this conclusion is based on indirect evidence of HPV presence and on the concept of “field cancerization” [48].

Because ANXA1 is a substrate for protein kinase C (PKC) and for various tyrosine kinases such as EGFR [49], it is subject to phosphorylation of amino acid residues, a process that can confer various biological activities on the protein [28,29]. PKC executes the phosphorylation of ANXA1 in the serine 27 residue (ANXA1-SER), which is linked to the anti-inflammatory effects of the protein [30]. A study using an experimental model of endotoxemia induced by bacterial lipopolysaccharides demonstrated an early rise in the level of ANXA1-SER in response to inflammatory stimuli [22]. In contrast, phosphorylation of tyrosine 21 (ANXA1-TYR) by EGFR and by other tyrosine kinases is related to the process of cell proliferation. A study showed that both the expression of ANXA1 and the phosphorylation of the protein at tyrosine 21 are increased in the liver during the development of mouse embryos and decreases after birth. It returns to an elevated state following partial hepatectomia [31]. These changes suggest that a relationship exists between tyrosine 21 phosphorylation, proliferation of hepatocytes and regeneration of the liver. Moreover, studies have demonstrated a co-localization between ANXA1 and of EGFR both in the cell membrane and in the cytoplasm [50], again attesting to an interaction between these two markers.

In the present study, we determined that the expression of ANXA1-SER was increased in the normal epithelia compared to carcinomas in the HPV + cases. This difference was not observed in the HPV- cases. The increased expression in the normal HPV + tissue compared to in the HPV + tumors may indicate a physiological response to the inflammatory process generated by the viral infection. This response could be lost in cancer through unknown mechanisms that inactivate this regulatory pathway. Another possible explanation is the capacity of the tumor to escape antitumor immunological mechanisms (immune evasion), with a consequent reduction in immunological and inflammatory aggression and in the need to modulate this inflammation. The immune evasion process is well known in various neoplasms [51] including cancers of the head and neck [52]. The absence of an increase in ANXA1-SER in the HPV- epithelium samples compared to the HPV- carcinoma tissues probably arises from the absence of an inflammatory reaction to the viral infection.

We observed a reduction in the expression of ANXA1-TYR in cancers compared to the epithelia in both the HPV + and HPV- samples, a finding that contrasts with the data reported in a recent study of squamous cell carcinoma of the cervix [27]. The reduction in ANXA1-TYR in oropharyngeal cancer was independent of HPV status, which may reflect the use of the protein by EGFR in the process of inducing cell proliferation independent of whether the etiology of the tumor involves the virus or not. During signaling for cell proliferation, EGFR is known to become internalized into the cell, where it interacts with and phosphorylates ANXA1 to produce ANXA1-TYR; the resulting EGFR-ANXA1-TYR complex subsequently translocates to multivesicular bodies [26]. We recognize that further studies are needed to explore these findings.

To better understand both the participation of p53 in carcinogenesis of the oropharynx and the relationship between p53 and ANXA1 and HPV, we evaluated p53 expression by immunofluorescence. Nuclear positivity for p53 staining denotes mutation of the TP53 gene or other changes that inactivate and stabilize the protein [53]. Such immunostaining is generally identified only in cancers or dysplastic tissues [18,54]. Studies have indicated the existence of a binding site for p53 in the promoter for the *ANXA1* gene, inducing its expression [55]. Our study, however, did not indicate an increase in the expression of ANXA1 in the p53+ samples compared to in the p53- ones.

However, we did observe a reduction in the expression of p53 in HPV + tumors compared to in HPV- ones. The E6 and E7 oncoproteins produced by high-risk serotypes of the HPV virus can inactivate the molecular mechanisms that control the cell cycle and apoptosis. Specifically, the E6 protein is able to degrade p53 and to interact with the pro-apoptotic proteins Bax and Bak, inactivating these mechanisms of protection against carcinogenesis [56]. Therefore, the presence of low levels of p53 in the HPV + tumors might mean either that an alternative mechanism that does not involve mutation exists for inactivating p53 in these tumors or that the protein is hypoexpressed even in tumors with mutation. In the latter case, the mutated p53 could possibly have been degraded and, therefore, did not accumulate in the cells. Westra *et al.* observed a reduction in the occurrence of mutations in the *p53* gene in HPV + tumors of the head and neck. In addition, they demonstrated that mutations that were found did not affect the transactivation site of the p53 protein, suggesting that carcinogenesis mediated by HPV is independent of p53 mutation, even in cases in which a mutation is present [57].

## Conclusions

Our findings confirm the presence of the HPV genome in a substantial proportion of oropharyngeal tumors, as well as a reduction in the expression of p53 in HPV + tumors. We also observed a reduction in the expression of ANXA1 in these tumors in comparison to non-neoplastic epithelia. We did not observe a relationship between the expression of ANXA1 or its phosphorylated forms and the presence of HPV in oropharyngeal cancer. However, our data suggest that, in non-neoplastic epithelium, ANXA1 expression is increased in individuals whose tumor is positive for HPV. The importance of ANXA1 to cancers of the head and neck is becoming more apparent, and larger studies are essential to increase our understanding of the participation of this protein in oropharyngeal carcinogenesis.

## Methods

### Patients

We obtained samples from 22 patients with previously untreated oropharyngeal cancer diagnosed at Mato Grosso Cancer Hospital – Cuiabá/Brazil. The samples were derived from tumor biopsies and from products of surgical resections of tumors with non-neoplastic epithelium at the tumor margins. These samples were used to make comparisons between tumor and non-tumor tissues. In addition, we used 5 samples of epithelial tissue obtained from the oropharynx of people who had died from other causes and who did not have head or neck cancers. We asked all of the study participants or their family members to read and sign a free and clarified consent form. The study was approved by the Ethics Committee of the Federal University of Mato Grosso (Case 973/CEP-HUJM/2010).

### Histopathological analysis

We prepared 3-μm sections of each sample. The slices were deparaffinized with xylene, hydrated with alcohol solutions of decreasing concentrations and stained with hematoxylin-eosin. A medical pathologist then analyzed the material to confirm the presence of normal epithelium and/or neoplasia and to evaluate the grade of histological differentiation of each tumor according to the World Health Organization classification system [58].

### Determination of the presence of HPV DNA via chromogenic in situ hybridization (CISH)

To analyze the presence of HPV genetic material, we used the Rembrandt® hybridization and detection kit (PanPath, Budel, Netherlands) according to the manufacturer’s instructions. This kit includes two sets of probe mixtures. One can identify the presence of HPV serotypes 16 and 18, while the other one targets serotypes 31 and 33. Formalin-fixed, paraffin-embedded samples were sectioned into 4-μm slices with a Hyrax M60 microtome (Zeiss, Germany) and heated at 56-60°C for 2 h. Next, we deparaffinized the sections in xylene (2 baths), dehydrated them in 100% ethanol for 5 minutes, and dried them at room temperature. The slides underwent proteolytic treatment with diluted pepsin in 0.1 N HCl (1:100) for 30 minutes, followed by dehydration in rising concentrations of ethanol. The denaturation step involved applying 20 μL of probe solution to each slice, covering the slices with coverslips sealed with silicon, and heating them at 95°C for 5 minutes. Next, we incubated the material at 37°C in a wet chamber for 12 to 16 hours to allow hybridization to occur. We then removed the coverslips by immersing the slides in tris-buffered saline (TBS). We performed the detection step by incubating the slides in a solution containing horseradish peroxidase (HRP) for 30 minutes and then amplifying the signal by incubation with a 3-amino 9-ethylcarbazol (AEC) substrate for 5 to 15 minutes. Finally, we counter-dyed the slides with Methyl Green. The same reactions were performed with a cervical cancer cell line (CasKi) that contains the HPV 16 genome, provided by the manufacturer, as a positive control.

### Immunofluorescence analysis of the protein expression of p16, p53, ANXA1, ANXA1-SER, and ANXA1-TYR

We obtained histological sections of 3-μm thickness and placed them onto slides with a biological adhesive. After deparaffinization with xylene, we hydrated the sections in solutions containing decreasing concentrations of alcohol and incubated them in a bain-marie at 70°C in a 0.21% sodium citrate solution (pH = 6.0) for 1 hour. Next, we treated the cuts with 3% hydrogen peroxide in 70% methanol for 30 minutes to block tissue peroxidase. To induce permeabilization, we incubated the slides in 0.4% Tween 20 in PBS for 15 minutes and blocked them for 30 minutes with 5% bovine serum albumin (BSA) (Sigma-Aldrich, Rio de Janeiro, Brazil), diluted in PBS. The samples from each patient were then incubated with the following primary antibodies in 1% BSA for 18 hours at 4°C in a humidified chamber: mouse anti-p16 (Santa Cruz Biotechnology, Santa Cruz, CA, USA) at a dilution of 1:200; rabbit anti-ANXA1 (Invitrogen, Frederick, MD, USA) and mouse anti-p53 (Invitrogen, Frederick, MD, USA) together at a dilution of 1:200 for each antibody (this anti-p53 antibody is capable of identifying both the wild-type and the mutated forms of the protein); rabbit anti-ANXA1-SER27 and anti-ANXA1-TYR21 at a dilution of 1:100 for each antibody [31,59]. Next, the samples were incubated in secondary goat anti-rabbit antibodies (for ANXA1 and its phosphorylated forms) and anti-mouse (for p16 and the dual-stained slides with p53) together with the fluorochromes ALEXA FLUOR 488 and ALEXA FLUOR 546 (Invitrogen, Eugene, OR, USA) to label the rabbit and mouse antibodies, respectively (dilution of 1:50 in 1% BSA for 1 hour at room temperature in a humidified chamber). We also added the nuclear fluorescent dye 4′,6-diamidino-2-phenylindole (DAPI), to facilitate the morphological identification of the tissues. The slides were washed in PBS and mounted in glycerin with PBS (in a 1:1 ratio). We identified the immunostained cells of the squamous cell carcinoma fragments and the non-neoplastic epithelium using an AxioScope.A1 microscope (Carl Zeiss, Germany). We quantified the immunoexpression of the ANXA1, ANXA1-SER, and ANXA1-TYR proteins by median optical density (MOD) with the aid of image analysis software (Axiovision v. 4.8.1, 2009) using arbitrary units (a.u.) ranging from 0 to 255. Expression of p16 and p53 was scored as positive or negative. We compared the expression of ANXA1 in tissues with and without p53 positivity. For densitometry measures, images were obtained with a 20× objective lens and are presented as the average ± SEM based on readings from 5 random sample areas including both the central non-necrotic area of the tumor and the edge of the tumor invasion.

### Statistical analyses

We compared the averages for the various fluorescent densities using one-way ANOVA with Bonferroni’s post-test. We used the *χ*^2^ method to compare the proportion of tissues with p53 expression between the HPV + and HPV- groups. We used GraphPad Prism v. 5.01 for Windows (La Jolla, CA, USA) to perform the analyses. *P*-values below 0.05 were considered to be significant.

## Competing interests

All authors involved in this research have no competing interests to declare.

## Authors’ contributions

CQ was responsible for sample collection, for performing all experiments, for data analysis and for the elaboration of the manuscript. CN did the pathological review of all samples. ES gave advice for the ANXA1 techniques and provided the ANXA1 antibodies. AD designed the study, supervised all the experiments, did the statistical analysis and collaborated with the manuscript. All authors read and approved the final manuscript.
